# Growing Teratoma Syndrome: A Rare Case Report and Review of the Literature

**DOI:** 10.1155/2012/134032

**Published:** 2012-05-08

**Authors:** Nirmala Kampan, Trika Irianta, Arifuddin Djuana, Lim Pei Shan, Mohd Hashim Omar, Ahmad Zailani Hatta Mohd Dali

**Affiliations:** ^1^Obstetrics & Gynaecology, UKM Medical Centre, 56000 Cheras, Kuala Lumpur, Malaysia; ^2^Bagian Obstetri dan Ginekologi, Universitas Hasanuddin, Makassar 90245, Indonesia

## Abstract

Growing teratoma syndrome is rare and usually it occurs in the younger aged group. The use of chemotherapy following initial surgical resection will yield the diagnosis following tumour enlargement. Complete resection is usually curative and renders better prognosis.

## 1. Introduction

This report concerned a young patient with growing teratoma syndrome who required complete resection. Residual disease is the commonest suspicion following fertility conserving surgery for immature teratoma of ovary in a young woman. Administration of adjuvant chemotherapy is the usual course pathway for management of residual disease. An enlarging intraperitoneal mass despite course of chemotherapy is usually due to treatment failure but rarely may be as a result of growing teratoma syndrome. Complete resection is essential to prevent progression of tumour and is often curative, hence, it will render better prognosis as mature teratoma, are resistant to both chemotherapy and radiotherapy.

## 2. Case Presentation

A 17-year-old, nonparous girl presented with a one-month history of noticeable pelvic mass and was diagnosed to have a left ovarian tumour in 2007 with a raised serum CA-125 of 350 IU/L. Other tumour markers were not performed due to financial constraint. An intraoperative frozen section revealed immature teratoma and she underwent fertility sparing surgery. A staging laparotomy with left salpingo-oophorectomy, peritoneal cytology, pelvic lymph nodes sampling, and infracolic omentectomy was performed. Postoperative histology report revealed mature teratoma with focal area of immature teratoma of the left ovary. There was no malignant cell infiltration to the omentum or lymph nodes. Histology revealed immature teratoma grade I, FIGO stage 1a. Despite being advised for close surveillance, she defaulted followup after 6 months postoperative.

One year later, she presented with lower abdominal discomfort and resought medical advice. She developed radiological recurrence of pelvic mass with a raised serum CA-125 of 180 IU/L following which she completed 6 courses of systemic chemotherapy (carboplatin-paclitaxel). After chemotherapy, the tumour marker normalised (<35 IU/L) but the pelvic mass progressively increased in size. CT scan of the abdomen and pelvis revealed presence of bilateral large adnexal masses with infiltration into uterus and also possibly to the sigmoid colon. Growing teratoma syndrome was suspected. She underwent staging laparotomy and complete excision of the tumour. Full bilateral pelvic lymphadenectomy was done. Postoperative CT scan of the abdomen and pelvis showed no residual disease. Histology of excised mass revealed mature teratoma with no presence of immature cells. The patient remained with no recurrence at the time of this report, which is 8 months after the second operation for GTS.

## 3. Discussion

Female ovarian growing teratoma syndrome (GTS), a rare encounter, is defined as an enlarging mature teratoma that arises during or following chemotherapy for a malignant germ cell tumor [1]. Five years later, it was renamed as “chemotherapeutic retroconversion.” The pathogenesis of this rare occurrence remained debatable. Selective elimination of the malignant cells by chemotherapeutic agents or differentiation of malignant cells into mature teratoma components following exposure to chemotherapeutic agents may be the two possible mechanisms responsible for development of GTS. As a result, often this is misinterpreted as chemo-resistant tumour or recurrence. Complete resection is the key to a longer remission, however, the dilemma arises usually in younger women. Very few cases are reported on young Asian women with management still being debatable [2–7].

The youngest age reported in the literature is 5 years old [3]. The initial presenting symptoms are usually abdominal distension or discomfort. The initial histopathology is usually immature teratoma. As GTS mainly occurs in the young patient or with fertility concern, the initial surgery they usually underwent unilateral salpingo-oophorectomy. It is noted that the decision for lymphadenectomy is done not in all patient but it has been reported to have better local control and lowered the disease progression to GTS. The development of GTS had been reported as early as 3 months and in some cases, delayed till 8 years. Monitoring of response to chemotherapy is by serum tumour markers till low or normal levels [2–18]. This is one of the first reported growing teratoma from a completely excised stage 1a immature teratomas. The possible explanation for the appearance of growing teratoma syndrome may be as a result of micrometastases of the remaining immature teratoma cells within the peritoneal cavity. This may be as a result of intra-abdominal dissemination despite intact capsule which may occur spontaneously preoperatively.

Despite normalization of serum tumour markers during chemotherapy, the metastatic tumour grows and is usually identified on radiological imaging or ultrasonography. The feature usually reported in literature is soft pelvic tissue mass on CT scan or echogenic mass on ultrasound pelvis. Complete surgical excision is the treatment of choice with retroperitoneal pelvic and para-aortic lymph node dissection [2–12, 14–17, 19]. Most patient had laparotomy excision of tumour mass rather than by laparoscopy. This is probably due to adequacy concern of the selected method. Patients who had complete excision had longer disease-free interval up to 8 years with lowered recurrence episodes [6]. GTS varied in growth rate and is chemoresistant in most cases [2, 3, 11–13]. Early decision for complete resection had lowered morbidity in this patient as with delay, GTS that can grow rapidly may encase the blood vessels and other vital structures leading to pressure effect and potential risk of vascular thrombosis, ureteral obstruction, bowel obstruction, or colonic fistula [1–9, 12, 16–19]. The majority of mortality of GTS is related to postoperative complications [18]. Malignant transformation has been reported up to 3% of cases [16, 17]. The predetermining factors for development of GTS were reported as incomplete resection of primary tumour, presence of mature teratoma cells in the initial histology and no reduction in the size of tumour after chemotherapy [12].

GTS has an overall good prognosis with only a few reported deaths [4, 5, 18, 19]. The 5-year overall survival rate of patients who had underwent surgery following GTS is 89% [18]. However, close regular followup is essential as recurrence may ensue up to the 10 years after the initial diagnosis [6, 12]. GTS had been reported to have even occurred following a successful pregnancy [6].

##  Conflict of Interests

The authors declare that there are no conflict of interests.
